# A Novel Lactose/MCC/L-HPC Triple-Based Co-Processed Excipients with Improved Tableting Performance Designed for Metoclopramide Orally Disintegrating Tablets

**DOI:** 10.3390/pharmaceutics16070959

**Published:** 2024-07-19

**Authors:** Xiaorong Dai, Jiamin Wang, Bo Yan, Qian Wang, Yan Shen, Yongkang Chen, Yu Tian

**Affiliations:** 1 Department of Gastroenterology, Taixing People’s Hospital, No. 1 Changzheng Road, Taixing 225400, China; 2 Center for Research Development and Evaluation of Pharmaceutical Excipients and Generic Drugs, School of Pharmacy, China Pharmaceutical University, 24 Tong Jia Xiang, Nanjing 210009, China; 3 Department of Pharmacy, Taixing People’s Hospital, No. 1 Changzheng Road, Taixing 225400, China; 4 School of Medicine, Shanghai University, Shanghai 200444, China; 5 Institute of Geriatrics (Shanghai University), Affiliated Nantong Hospital of Shanghai University (The Sixth People’s Hospital of Nantong), School of Medicine, Shanghai University, Nantong 201613, China

**Keywords:** co-processed excipients, metoclopramide, orally disintegrating tablets, pharmacokinetics

## Abstract

New co-processed excipients comprising lactose (filler and sweetener), microcrystalline cellulose (MCC, filler), and low-substituted hydroxypropyl cellulose (L-HPC, disintegrant and binder) were developed via solvent evaporation for the preparation of metoclopramide orally disintegrating tablets (MCP ODTs). Single-factor and Box–Behnken experimental designs were employed to optimize the formulation. The optimized formulation ratios were water: MCC: lactose (g/g) = 17.26:2.79:4.54:1. The results demonstrated that particles formed by solvent evaporation had superior flowability and compressibility compared to the physical mixture. Tablets compressed with these co-processed excipients exhibited a significantly reduced disintegration time of less than 25 s and achieved complete dissolution within 5 min. Pharmacokinetic studies revealed that MCP ODTs significantly improved C_max_, which was 1.60-fold higher compared to conventional tablets. In summary, the lactose/L-HPC/MCC triple-based co-processed excipients developed in this study are promising and could be successfully utilized in orally disintegrating and fast-release tablets.

## 1. Introduction

Orally disintegrating tablets (ODTs) are designed to dissolve in the mouth within a few seconds upon contact with saliva, eliminating the need for water [1]. Features like quick dispersion, rapid onset of action, ease of swallowing, and the ability to be taken without water make ODTs ideal drug delivery systems for patients with special needs [2]. These tablets are especially beneficial for elderly patients affected by dysphagia or paralysis, as well as infants and young children, due to their high patient compliance [3].

Currently available ODTs are typically produced through methods such as molding, mass extrusion, freeze-drying, spray-drying, and direct compression [1]. Due to its ease of large-scale production, simplified and low-cost process, and the absence of requirement for specialized equipment, direct compression becomes a preferred method for ODTs production in pharmaceutical industries [4]. However, the direct compression method necessitates excipients exhibiting good flowability and compressibility, which can be challenging for most currently available excipients [5]. Single-component excipients in particular often fail to meet the necessary requirements for direct compression of ODTs. Therefore, multifunctional co-processed excipients with enhanced properties are essential to enable feasible direct compression and improve the overall performance and characteristics of ODTs [6].

Co-processed excipients have emerged as a promising method for enhancing the properties of excipients used in direct compression. This method is more time- and cost-efficient than developing excipients with entirely new chemical structures. Unlike a simple mixture of several excipients [7], co-processed excipients are meticulously designed and produced through a series of physical co-processing steps [8]. Consequently, the most significant advantage of co-processed excipients is their superior compressibility and flowability compared to simple physical mixtures, thanks to the controlled and optimal particle size and distribution [9]. This leads to consistent tablet weight and uniformity. Additionally, co-processing of excipients improves the disintegration time and dissolution rate of ODTs due to the increase of porosity, ensuring the tablets quickly break down in the mouth without the need of water [10]. Moreover, tablets made with co-processed excipients generally exhibit higher mechanical strength, reducing friability and enhancing durability of the tablets during handling and transportation [11]. Co-processed excipients have also been reported to decrease grittiness while improving the palatability and mouthfeel of ODTs for children and the elderly [5,12]. Overall, co-processed excipients are designed to tackle multiple formulation and manufacturing challenges associated with ODTs, thereby enhancing their performance and patient acceptability [13].

Proper selection of suitable excipients based on their required functions and material properties is crucial in the formulation process. Once the appropriate excipients are chosen, determining the correct proportion of each component is essential to achieve the desired characteristics. Additionally, an effective manufacturing process for co-processed excipients is vital for optimizing co-processing. Currently, common co-processing methods include co-transformation, co-milling, co-precipitation, and co-crystallization [14,15]. These operations enable excipient particles to interact at the sub-particle level [16], providing an effective tool for developing excipients with enhanced functions. The commonly co-processed excipients used in ODTs typically consist of two or three components, including disintegrant, filler, binder, and lubricant [17]. Besides these, co-processing generally involves one plastic excipient and one brittle excipient. Maarschalk reports on co-processing that uses a large amount of brittle material and a small amount of plastic material, such as in cellactose, which consists of 75% lactose (brittle) and 25% cellulose (plastic) [18]. This specific combination prevents excessive elastic energy from being stored during compression, resulting in minimal stress relaxation and a reduced tendency for capping and laminating [19]. However, other extreme combinations exist, such as silicified microcrystalline cellulose (SMCC), which contains a large amount of microcrystalline cellulose (MCC, plastic) and a small amount of silica (brittle) [20]. These examples demonstrate that co-processing typically involves materials with both plastic deformation and brittle fracture characteristics, which are essential for optimal tableting performance.

MCC is a rod-shaped or granular crystal produced by hydrolyzing natural fibers with strong acid under heated conditions. It features a spongy, porous structure. Under pressure, the disordered porous structure of MCC becomes linearly arranged and undergoes plastic deformation, enabling water molecules to enter the tablet, break the hydrogen bonds between microcrystals, and promote rapid disintegration [21]. The small, low-density MCC particles lead to a higher proportion in formulations for the same mass, resulting in tablets with low actual density and high porosity. This allows water to quickly enter the core and accelerate disintegration. MCC has good compressibility and is suitable for direct compression methods. However, due to its weak swelling properties, it is generally not used alone as a disintegrant but often combined with other excipients with strong swelling properties, such as low-substituted hydroxypropyl cellulose (L-HPC) [22]. L-HPC is a long fibrous powder with rough and uneven surface structures and a large specific surface area. When mixed and compressed with other excipients or drugs, it forms voids and capillaries within the tablet core. L-HPC’s high porosity accelerates water absorption, allowing the tablet to disintegrate rapidly. Additionally, the rough surface structure of L-HPC particles leads to substantial interlocking between particles during compression, enhancing the adhesion between the drug and the particles. This contributes to excellent compressibility, strong hardness, and tablets with improved gloss and appearance [23]. Consequently, L-HPC is frequently employed as a binder in solid dosage forms. Lactose is available as white to off-white crystalline granules or powder. It is tasteless with a slight sweetness. The stable crystalline forms of lactose are α-lactose monohydrate, β-anhydrous lactose, and stable α-anhydrous lactose. The sweetness of α-lactose is about 20% that of sucrose, while β-lactose is 40%. Lactose is stable, non-hygroscopic, and has good compressibility. It is compatible with most drugs, making it widely used as a filler and diluent in tablets and capsules.

Metoclopramide, classified as a BCS III drug [24], is a white crystalline powder that is odorless, bitter, and nearly insoluble in water [25]. Metoclopramide ODTs were approved by the FDA (METOZOLV^TM^ ODT) for short-term treatment of patients with acute and recurrent diabetic gastroparesis and symptomatic gastroesophageal reflux disease. These ODTs offer a valuable option for individuals who have difficulty swallowing tablets or capsules due to conditions such as odynophagia, nausea, vomiting, dysphagia, and heartburn, or in situations where oral administration is challenging [26]. However, the excipients used in commercial metoclopramide ODTs are not co-processed, and the preparation method is freeze-drying [27], which is complex, requires specialized equipment, and is costly. Therefore, developing new co-processed excipients that combine various properties of excipients can enhance the performance of ODTs, including flowability, disintegration time, and dissolution stability. This approach is suitable for direct compression, which could simplify the production of metoclopramide ODTs, reduce costs, and improve patient compliance.

The objective of this study was to develop a novel co-processed excipient, comprising lactose, low-substituted hydroxypropyl cellulose (L-HPC), and MCC, suitable for orally disintegrating tablets (ODTs) through an appropriate formulation process. Metoclopramide, chosen as the model drug due to its poor solubility, was incorporated into ODTs using these co-processed excipients via direct compression. Lactose was selected as a brittle excipient due to its predominant brittle deformation upon compression. However, lactose alone often lacks optimal compressibility as a filler. In contrast, MCC, characterized by its sponge-like porous structure, primarily undergoes plastic deformation after compression, providing excellent compressibility and earning the designation of a “dry adhesive” for direct compression processes [28]. L-HPC, with its high hygroscopicity and favorable swelling properties in water, contributes to the linear arrangement of MCC’s porous structure when combined and compressed. Moreover, the interaction between MCC and water facilitates rapid tablet disintegration by disrupting hydrogen bonds between crystallites within the tablet core.

In this research, single-factor and Box–Behnken experiment designs were utilized to obtain the optimal formulation of co-processed excipients. Subsequently, MCP ODTs based on this co-processed excipient technology were prepared and characterized. Furthermore, both in vitro dissolution and in vivo pharmacokinetic studies of the metoclopramide ODTs were conducted.

## 2. Materials and Methods

### 2.1. Materials

Microcrystalline cellulose (MCC (SH101), low-substituted hydroxypropyl cellulose (L-HPC (LH-22, LH-21)), and magnesium stearate were kindly provided by Anhui Shanhe Pharmaceutical Excipient Co., Ltd. (Huainan, China). Lactose was obtained as a gift sample from Dawning Pharmaceutical Co., Ltd. (Changzhou, China). Menthol was purchased from Shanghai Baichun Pharmaceutical Co., Ltd. (Shanghai, China). Sucralose was obtained from Jiangxi Alpha Hi-tech Pharmaceutical Co., Ltd. (Pingxiang, China), and metoclopramide was obtained from Liaoyuan Yinying Pharmaceutical Co., Ltd. (Liaoyuan, China). Commercial metoclopramide tablet (ANI Pharmaceuticals Inc., Baudette, MN, USA) has been used as the reference.

### 2.2. Formulation Optimization

#### 2.2.1. Effects on Various Fillers

To produce co-processed excipients using MCC and L-HPC, different ratios of mannitol, maltitol, sorbitol, and lactose were employed as fillers. The fluidity test was utilized to assess the quality of the co-processed excipients.

#### 2.2.2. Effects on Various Disintegrants

In this study, varying proportions of L-HPC or sodium carboxymethyl starch (CMS-Na) were utilized as disintegrating agents to create co-processed excipients with MCC and lactose. The fluidity investigation was employed to assess the quality of the co-processed excipients.

#### 2.2.3. Effects on Different Preparation Methods

This experiment investigated the impact of heat treatment time on the preparation of co-processed excipients using L-HPC, lactose, and MCC. Heat treatment time and heat treatment temperature were chosen as the single-factor screening indicator for the preparation of co-processed excipients, and fluidity measurements were taken to evaluate their quality.

#### 2.2.4. Box–Behnken Experiment Design

Based on the results of single-factor experiments, a Box–Behnken response surface experimental design method with three factors and three levels was employed (refer to Table S1). The investigation factors included the ratios of water to L-HPC (*w*/*w*), MCC to L-HPC (*w*/*w*), and L-HPC to lactose (*w*/*w*), while fluidity (measured by the angle of repose and Carr’s index) and disintegration time were chosen as response values to optimize the formulation of the co-processed excipients.

### 2.3. Preparation of Co-Processed Excipients

The co-processed excipients comprising lactose, L-HPC, and MCC were prepared using the wet-method solvent evaporation technique. Initially, L-HPC was dissolved in a specified volume of water at 80 °C for 15 min. Subsequently, lactose was added and stirred using electromagnetic heating at 80 °C for 3 h. Following this, MCC was gradually incorporated, and the mixture was stirred at room temperature for an additional 3 h. The resulting mixture was then dried at 40 °C, pulverized for 1 min using a high-speed stirrer, and sieved through an 80-mesh screen.

### 2.4. Characterization of Co-Processed Excipients

#### 2.4.1. Flowability

##### The Angle of Repose

The angle of repose was determined using the fixed funnel method [29]. The funnel was securely positioned on a water platform, and the material was dispersed through a 14-mesh screen. Materials were added through the funnel until a cone-shaped stack was formed. The angle of repose was then measured using the intelligent powder property tester (BT-1001, Dandong Bettersize Instrument Co., Ltd., Dandong, China). All experiments were conducted in triplicate.

##### Bulk Density and Tapped Density

Compressibility, as indicated by bulk density (ρ_b_) and tapped density (ρ_t_), was also utilized to assess flowability. Carr’s index and the Hausner ratio were employed to evaluate compressibility of the powders [8]. Co-processed particles were poured into a cylinder (pre-weighed as W_0_) through a vibrating funnel until full. The samples were then weighed with the cylinder (W_1_), and the volume was adjusted to 100 cm^3^. Subsequently, the cylinder was tapped at least 3000 times until the sample volumes no longer decreased, and the final volume (V, cm^3^) was recorded. ρ_b_ and ρ_t_ were calculated using Equations (1) and (2):

(1) $$\rho_{b}=\frac{W_{1}-W_{0}}{100}$$

(2) $$\rho_{t}=\frac{W_{1}-W_{0}}{V}$$

The Carr’s index and Hausner ratio were then calculated according to Equations (3) and (4):

(3) $$\mathrm{Carr’s}\;\mathrm{index}=\frac{\rho_{t}-\rho_{b}}{\rho_{t}}\times 100\%$$

(4) $$Hausner\;ratio=\frac{\rho_{t}}{\rho_{b}}$$

#### 2.4.2. Compactibility

Tensile strength was utilized to evaluate the compactibility of the co-processed particles. The co-processed particles were compressed using a single-punch tablet machine (DP-30, Shanghai TianFan Instrument Co., Ltd, Shanghai, China) equipped with 7.0 mm round flat-faced tooling and subjected to various pressures. The weight of the compacts was maintained at 135 mg. Measurements of thickness (H, mm), diameter (D, mm), and breaking force (Fc, N) were taken 24 h after compression at each pressure. Tensile strength (σ_T_) was determined using Equation (5):

(5) $$\sigma_{T}=\frac{2F_{c}}{\pi HD}$$

#### 2.4.3. Hygroscopicity

The hygroscopicity was assessed using a series of closed dryers containing different saturated inorganic salt solutions to mimic stable relative humidity (RH) environments at 25 °C [7] (Table S2).

#### 2.4.4. Surface Morphology and Physical States

The morphology of the materials was examined using a scanning electron microscope (SEM, Hitachi SU8010, Hitachi Limited, Tokyo, Japan). The particles were dispersed onto a double-sided adhesive tape affixed to a silicon stub. Subsequently, the particles were sputter-coated with gold using a Hitachi ion sputter coater (E1010, Hitachi Limited, Japan) and observed at an acceleration voltage of 15 kV.

Infrared (IR) patterns were analyzed using an infrared spectrophotometer (NICOLET iS10, Thermo Fisher Scientific, Waltham, MA, USA). Materials were mixed with potassium bromide at a mass ratio of 1:50 and pressed to prepare the testing samples. All samples were scanned within the range of 500 to 4000 cm^−1^.

X-ray diffraction (XRD) patterns were examined using an X-ray powder diffractometer (D8 Advance, Bruker Corporation, Karlsruhe, Germany). The particles were loaded onto a horizontal square recess of the sample holder, and a razor blade was used to evenly spread the powder sample before scanning. The scan was conducted at a rate of 2.00 °/min over a 2θ range of 3.0–40.0°.

Approximately 5 mg of the sample was tested in a platinum crucible, with the temperature programmed at 10 °C/min, and thermogravimetric analysis (TGA) maps were recorded within the temperature range of 25–500 °C (TGA550, TA Instruments-Waters LLC, Austin, TX, USA).

Approximately 3 mg of the sample was sealed in an aluminum dry pot, with the temperature programmed at 10 °C/min under a nitrogen atmosphere (50 mL/min for purging and 100 mL/min for protective flux), and differential scanning calorimetry (DSC) curves were recorded within the temperature range of 25 to 300 °C (DSC250, TA Instruments-Waters LLC, USA). The instrument was calibrated with standard metals having a melting point close to the required temperature range, specifically indium (156.6 °C) and tin (231.9 °C).

### 2.5. Preparation of MCP ODTs

MCP ODTs were prepared using the direct compression method. Following the formulation of the commercial metoclopramide tablet, each tablet contained 5 mg of metoclopramide. A specific quantity of co-processed excipients (consisting of lactose, L-HPC, and MCC), along with the flavoring agent (0.3% *w*/*w*, sucralose = 1:7) and lubricant (0.5% *w*/*w*, magnesium stearate), were blended with metoclopramide. Subsequently, a single-punch tablet machine (DP-30, Shanghai Tianfan Pharmaceutical Machinery Factory, Shanghai, China) was employed to directly compress the powder formulation, resulting in MCP ODTs. The tablet weight was maintained at 135 ± 3 mg, and the compression pressure was regulated at 40 ± 10 N, as measured by a hardness tester (YD-35, Shanghai Tianfan Pharmaceutical Machinery Factory, China).

### 2.6. Characterization of MCP ODTs

#### 2.6.1. Disintegration Time

**Figure 1:**
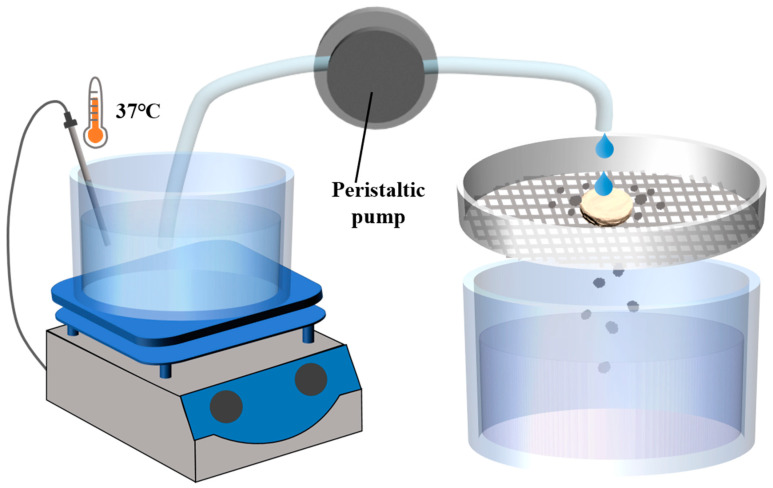
Schematic diagram of disintegrating equipment of disintegrating method.

According to the issued Guidance for Industry of Orally Disintegrating Tablets [30], the FDA specifically recommends that ODTs be considered solid oral preparations that disintegrate rapidly in the oral cavity, with an in vitro disintegration time of approximately 30 s or less according to the United States Phamacopeia (USP) disintegration method or alternative. According to the regulation, a disintegration device was designed [31]. The device primarily consists of an electronic heating magnetic stirrer, a 30-mesh screen, a constant flow peristaltic pump, and a liquid receiving device (Figure 1). The water in the electronic heating magnetic stirrer was heated to 37 °C, with the droplet flow rate adjusted to 2 mL/min and the vertical distance from the droplet source to the tablet set at 2 cm. The tablet was placed on the sieve, and timing begins when the water droplets start dripping onto the tablet. Timing stops when the tablet completely dissolves and passes through the screen. The elapsed time was recorded as the disintegration time (details on the resolution, correlation, and reproducibility of the device methods are provided in Supplementary Materials Tables S6–S8).

#### 2.6.2. In Vitro Dissolution

According to the United States Phamacopeia (USP), the in vitro dissolution of MCP ODTs was measured using the rotating basket method with a dissolution tester (RC806D, Tianjin Tianda Tianfa Instruments, Tianjin, China). Briefly, 900 mL of hydrochloric acid (pH 1.2) per vessel served as the dissolution medium, maintained at 37 (±0.5) °C throughout the experiment. The MCP ODTs were placed in the basket and dissolved at a speed of 50 rpm (*n* = 6). Samples of the dissolution medium will be extracted at 1, 3, 5, 10, 15, 20, 30, and 45 min after the start of dissolution. The content of metoclopramide in the filtered samples was analyzed by the HPLC system (Ultimate3000, Thermo Fisher Scientific, USA) at a wavelength of 275 nm. The mobile phase consists of 0.02 mol/L phosphoric acid (adjusted to pH 4.0 with triethylamine) and acetonitrile (81:19, *v*/*v*) at a flow rate of 1.0 mL/min. The chromatographic separations were performed on a C18 reverse column (4.6 mm × 250 mm, 5 μm) and the column was maintained at 25 ℃. Additionally, a precisely weighed metoclopramide reference substance was dissolved in the dissolution medium and quantitatively diluted to create a solution containing approximately 5.5 μg/mL, measured using the same method. Fresh medium at the same temperature and volume was replaced to the dissolution vessels immediately after each sampling. All experiments were conducted six times.

### 2.7. In Vivo Pharmacokinetics Study

All animal experiments were conducted in accordance with the guidelines of the National Health Guidance Association, and the experimental protocol was approved by the Ethics Committee of China Pharmaceutical University.

A randomized two-way crossover design (with a 7-day washout period) was used in the experiments. Six healthy beagle dogs were randomly assigned to groups A and B before dosing. MCP ODTs and Reglan^®^ were orally administered to groups A and B, with cross-administration in the second period. The dogs fasted for 12 h before administration, were allowed to drink water freely, and were fed four hours after administration. Blood samples of 1 mL were taken from the forelimb vein at specific times: 0 (pre-dose), 10, 20, 30, 45 min, 1, 1.5, 2, 3, 4, 6, 8, and 10 h on the days of oral administration. The collected blood was immediately transferred to Eppendorf tubes, centrifuged at 8000 rpm for 5 min, and the plasma was stored at −20 °C for further analysis.

After thawing at room temperature, 20 μL of sodium hydroxide solution (1 mol/L) and 20 μL of internal standard solution (tramadol hydrochloride, 15 μg/mL) were added to 300 μL of plasma and vortexed for 30 s. Then, 2 mL of dichloromethane was added and vortexed for 1 min. The mixture was centrifuged at 12,000 rpm for 10 min at 4 °C (KDC-140HR, Anhui USTC Zonkia Scientific Instrument Co., LTD, Hefei, China). A 1.5 mL portion of the lower organic phase was transferred to a centrifuge tube and evaporated to dryness in a vacuum concentrator (35 °C, 1500 rpm, 30 min) (ZL3-2K, Hunan Kecheng Instrument Equipment Co., LTD, Changsha, China). The residue was reconstituted with 100 μL of the mobile phase (0.02 mol/L phosphoric acid solution (pH adjusted to 4.0 with triethylamine)/acetonitrile = 81/19, *v*/*v*), vortexed for 1 min, centrifuged at 12,000 rpm for 10 min (HC-2062, Anhui USTC Zonkia Scientific Instrument Co., LTD, Hefei, China), and 20 μL of the supernatant was analyzed by HPLC. The HPLC system and mobile phase were the same as used in the dissolution studies, and the column temperature was set at 35 °C. The retention times of metoclopramide and tramadol hydrochloride (internal standard solution) were 12.85 and 17.87 min, respectively (Figures S1 and S2).

### 2.8. Statistical Analysis

A non-compartmental model analysis was performed using WinNonLin 7 software (Pharsight Corporation, Mountain View, CA, USA) to compare the pharmacokinetic parameters of MCP ODTs and Reglan^®^ in blood. Multivariate analysis of variance (ANOVA) was used to test for significance, with a two-sided test and 90% confidence interval employed to evaluate and determine the bioequivalence of the tablets. Data are expressed as the mean ± SD. A *p*-value of less than 0.05 (i.e., *p*  <  0.05) was considered statistically significant.

## 3. Results and Discussion

### 3.1. Optimized Formulation of Co-Processed Excipients

Table 1 demonstrates that lactose, mannitol, maltitol, and sorbitol were used as fillers in conjunction with L-HPC and MCC. The table also illustrates the fluidity and compressibility of the co-processed excipients. Flowability assessments involved measuring angle of repose, bulk, and tapped densities to determine the Carr’s index and Hausner ratio. Good flowability of a direct compression excipient is essential for producing tablets with uniform weight. When lactose and mannitol were utilized, the co-processed excipients exhibited an angle of repose less than 40°, indicating good fluidity and compressibility, making them potential tablet fillers [32]. Compressibility was evaluated using the Hausner ratio. A significant difference in compression was observed, with lactose showing the smallest compression, indicating superior compressibility and fluidity. Therefore, lactose is preferred as a filler for co-processing.

Table 2 reveals that co-processing different types of L-HPC and CMS-Na with lactose and MCC as disintegrants resulted in varying fluidity and compressibility of the co-processed excipients. When L-HPC (LH21, LH22) and CMS-Na (1) were used, the co-processed excipients exhibited an angle of repose less than 40°, meeting industrial production standards. Comparing compressibility (Carr’s index and Hausner ratio), L-HPC (LH21) showed the smallest compression, indicating good compressibility and fluidity. CMS-Na, characterized by its ellipsoid-like shape and uniform particle size distribution, exhibited good fluidity but lower compressibility compared to L-HPC. The higher degree of substitution in LH-21 compared to LH-22 results in greater swelling, higher hydration, and therefore better disintegration and promotion of dissolution. Consequently, L-HPC (LH21) is initially recommended as a disintegrating agent for co-processing.

As shown in Table 3 and Table 4, when L-HPC was heat-treated at 80 °C for 0.25 h, lactose at 80 °C for 3 h, and MCC at room temperature for 3 h, the co-processed excipient demonstrated good fluidity and compressibility. This is due to the fact that, at high temperatures in an aqueous solution, α-lactose monohydrate from lactose undergoes a variable optical rotation process, partially converting to β-anhydrous lactose, which has superior fluidity and compressibility compared to α-lactose [33].

### 3.2. Box–Behnken Experiment Design

**Figure 2:**
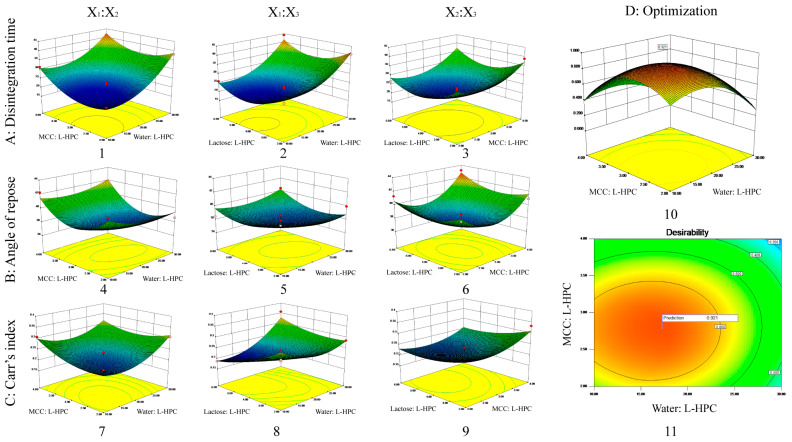
3D response surface plot showing influence of co-processed excipients on (**A**) disintegrating time (1–3); (**B**) angle of repose (4–6); (**C**) Carr’s index (7–9). The interaction effects of X_1_ and X_2_ (1, 4, 7); the interaction effects of X_1_ and X_3_ (2, 5, 8); the interaction effects of X_2_ and X_3_ (3, 6, 9); and the desirability 3D plot (10) and contour plot (11) for (**D**) optimized formulation.

According to the Box–Behnken design outlined in Table 5, 17 sample groups were prepared, and their fluidity (measured by angle of repose, bulk density, and tap density) was assessed. Additionally, MCP ODTs were prepared as described previously, and their disintegration time was measured as corresponding indicators. The results of these measurements are presented in Table 5 and Figure 2. Furthermore, the experimental data were analyzed, and the results of fitting the model were presented in Table 6. The nonlinear equations were observed to follow the quadratic polynomial, with correlation coefficients R^2^ of 0.9663, 0.8642, and 0.8611 for the disintegration time, angle of repose, and Carr’s index, respectively, suggesting that the established model has a robust correlation and strong ability to predict actual values.

The experimental results were further scrutinized through analysis of variance. The mathematical models for the three response values are as follows:

(6) $$\begin{array}{l} \mathrm{Disintegrating}\;\mathrm{time}\;(s)=93.86-1.19X_{1}-27.97X_{2}-15.42X_{3}-0.18X_{1}X_{2}+0.04X_{1}X_{3}\\ +0.40X_{2}X_{3}+0.06{X_{1}}^{2}+5.59{X_{2}}^{2}+1.65{X_{3}}^{2} \end{array}$$

(7) $$\begin{array}{l} \mathrm{Angle}\;\mathrm{of}\;\mathrm{repose}\;\left({}^{\circ}\right)=73.27-0.61X_{1}-16.10X_{2}-3.35X_{3}+0.05X_{1}X_{2}+0.03X_{1}X_{3}\\ +0.17X_{2}X_{3}+0.0079{X_{1}}^{2}+2.52{X_{2}}^{2}+0.30{X_{3}}^{2} \end{array}$$

(8) $$\begin{array}{l} \mathrm{Carr’s}\;\mathrm{index}\;(\%)=0.70-0.018X_{1}-0.11X_{2}-0.082X_{3}-0.0018X_{1}X_{2}+0.0021X_{1}X_{3}\\ -0.0031X_{2}X_{3}+0.00044{X_{1}}^{2}+0.030{X_{2}}^{2}+0.0048{X_{3}}^{2} \end{array}$$

#### 3.2.1. Disintegrating Time

By conducting an analysis of variance, it was determined that X_1_, X_2_, X_1_^2^, X_2_^2^, and X_3_^2^ significantly influenced the disintegration time across all variables (*p* < 0.01) (Table S3). From the disintegration time model Equation (6), it is evident that the factors affecting disintegration time were negative, meaning that increasing X_1_, X_2_, or X_3_ reduced disintegration time. The preparation of ODTs using MCC and L-HPC was influenced by the swelling of excipients and the interaction forces between particles. MCC and L-HPC have uneven surfaces, high interaction forces between particles, and high porosity, allowing water molecules to quickly penetrate the tablet through the pores, wetting and disintegrating the entire tablet. MCC has poor swelling properties, while L-HPC has high swelling properties, resulting in better disintegration when combined. However, L-HPC also has adhesive properties that enhance adhesion when its proportion is increased, thereby hindering disintegration. Increasing the amount of solvent water during co-processing ensures uniform dispersion of the materials during mixing, which could increase the contact area and resulted in a tighter combination. Therefore, increasing X_1_ (water: L-HPC, i.e., increasing water or decreasing L-HPC), X_2_ (MCC: L-HPC, i.e., increasing MCC or decreasing L-HPC), or X_3_ (lactose: L-HPC, i.e., increasing lactose or decreasing L-HPC) will reduce the disintegration time.

The 3D plots of the interaction effects on disintegration time are shown in Figure 2A. According to the 3D plot of the interaction effects between X_1_ and X_2_, the disintegration time initially decreased and then increased as X_2_ increased, when X_1_ was at a constant level. Similarly, when X_2_ was at a constant level, the disintegration time initially decreased and then increased as X_1_ increased. The 3D plot of the interaction effects between X_1_ and X_3_ indicated that the disintegration time initially decreased and then increased as X_3_ increased, when X_1_ was constant. Likewise, when X_3_ was constant, the disintegration time initially decreased and then increased as X_1_ increased. The 3D plot of the interaction effects between X_2_ and X_3_ shows that the disintegration time initially decreased and then increased as X_3_ increased, when X_2_ was constant. Similarly, when X_3_ was constant, the disintegration time initially decreased and then increased as X_2_ increased. There were no interaction effects between any two factors, which was consistent with the results of the variance analysis.

#### 3.2.2. Angle of Repose

The angle of repose for the 17 formulations ranged from 36° to 43° (Table 5). Among all variables influencing the angle of repose, X_2_^2^ and X_3_^2^ had significant effects (*p* < 0.05) (Table S4). According to the angle of repose model Equation (7), the factors affecting the angle of repose were negative, meaning that increasing X_1_, X_2_, or X_3_ will reduce the angle of repose. During co-processing, some amorphous lactose converts to crystalline lactose, which has better flow properties. The lactose particles adhere to the excipient surface, improving flowability. Additionally, increasing the amount of solvent water during co-processing ensures uniform dispersion of the materials during mixing, increases the contact area, and creates a tighter bond. After the solvent water evaporates, larger particles form, enhancing flowability. MCC has a spongy, porous structure that primarily undergoes plastic deformation under pressure, providing good flowability and compressibility. Therefore, increasing X_1_ (water: L-HPC, i.e., increasing water or decreasing L-HPC), X_2_ (MCC: L-HPC, i.e., increasing MCC or decreasing L-HPC), or X_3_ (lactose: L-HPC, i.e., increasing lactose or decreasing L-HPC) will reduce the angle of repose.

The 3D plots of the interaction effects on the angle of repose are shown in Figure 2B. Similar to the disintegration time results, there were no interaction effects between any two factors, consistent with the variance analysis results.

#### 3.2.3. Carr’s Index

The Carr’s index (%) among the 17 formulations ranged from 18% to 36% (Table 5). According to Table S5, X_1_, X_1_X_3_, and X_1_^2^ significantly influence the Carr’s index (*p* < 0.05) among all variables considered. From the Carr’s index model Equation (8), it is evident that the factors affecting the Carr’s index are negative, indicating that increasing X_1_, X_2_, or X_3_ will decrease the Carr’s index. During co-processing, as the conversion of amorphous lactose to crystalline lactose proceeds, this leads to better flow properties and compressibility. Additionally, MCC has the unique spongy and porous structure that primarily undergoes plastic deformation under pressure, contributing to its good compressibility. This improved compressibility leads to a reduced Carr’s index. Therefore, increasing X_1_ (water: L-HPC, i.e., increasing water or decreasing L-HPC), X_2_ (MCC: L-HPC, i.e., increasing MCC or decreasing L-HPC), or X_3_ (lactose: L-HPC, i.e., increasing lactose or decreasing L-HPC) will improve compressibility and reduce the Carr’s index.

The 3D plots of the interaction effects on the Carr’s index are presented in Figure 2C. Similar to the results for disintegration time and angle of repose, there were no interaction effects observed between any two factors, consistent with the results of the variance analysis.

#### 3.2.4. Optimal Formulation

The criteria for optimizing the formulation include achieving the shortest disintegration time, the minimum angle of repose, and the lowest Carr’s index. Based on these criteria, the response surface of the formulation optimization was visualized using 3D maps generated by the optimization function of Design-Expert 8.0 (Figure 2D). The optimal formulation identified through optimization (weight/weight) consisted of the following: water: L-HPC at 17.26, MCC: L-HPC at 2.79, and lactose: L-HPC at 4.54. The predicted desirability was 0.921, with a theoretical disintegration time of 19.34 s, an angle of repose of 37.87 degrees, and a Carr’s index of 20.97%.

Three batches of co-processed excipients were produced based on the optimized formulation (Table 7), resulting in a disintegration time of 22.76 ± 1.11 s, an angle of repose of 36.52 ± 1.32 degrees, and a Carr’s index of 20.43 ± 1.60%, aligning closely with the theoretical values optimized through the Box–Behnken experimental design.

### 3.3. Characterization of Optimized Co-Processed Particles

#### 3.3.1. Flowability

The flowability of drug powders is a critical attribute for solid dosage forms, influencing various aspects of drug production, processing, and quality control, and is particularly important for the viability of direct compression methods. Powder flowability is primarily assessed through methods that measure particle friction, such as the angle of repose, bulk density, tap density, and Carr’s index. Meeting production fluidity criteria typically involves an angle of repose of no more than 40°, a bulk density of at least 0.4 g/cm^3^, and a Carr’s index below 30% [32]. According to the data in Table 8, the angle of repose for co-processed excipients containing lactose, MCC, and L-HPC was 36.52°, indicating favorable flow characteristics. The Carr’s index of 18.75 and Hausner ratio of 1.23 further validated the excipients’ good flow properties, rendering them suitable for powder direct compression processes.

#### 3.3.2. Compactability

**Figure 3:**
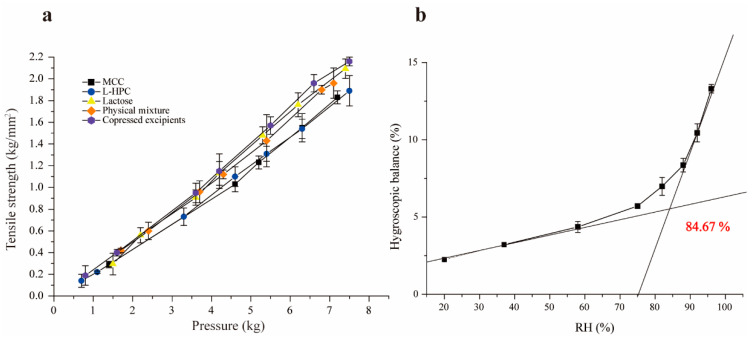
Tensile strength diagram of single excipients and co-processed excipients (**a**), and figure of critical relative humidity (**b**).

Tensile strength is a preferred measurement for assessing the compactability and formability of materials, commonly used in the quality evaluation and formulation screening of tablets [34]. Under identical pressure, the higher tensile strength indicates better compactability. The experimental results are presented in Figure 3a, which demonstrate that the tensile strength of all excipients increased with compression pressure, as expected. The co-processed excipients exhibited the highest tensile strength under the same pressure, indicating excellent compressibility and formability. This improvement is due to the combination of brittle lactose with the plasticity of MCC and L-HPC. When the pressure exceeds 4.5 kg, the tensile strength of lactose and co-processed excipients surpassed that of the physical mixture materials. While lactose has superior compactability compared to L-HPC and MCC, its compactability is slightly lower than that of the co-processed excipients, particularly at pressures over 4.5 kg. This is attributed to the transformation of α-lactose to β-lactose during co-processing, which enhances compactability. Additionally, since β-lactose lacks crystalline water, it is more brittle in its anhydrous form, leading to disruptions in the crystalline order and promoting crack formation [33,35].

#### 3.3.3. Hygroscopicity

Moisture absorption occurs when the partial pressure of water vapor in the air exceeds that of the saturated water vapor produced by the drug itself. This can lead to poor fluidity, reduced compressibility, and excessive impurities. At a given temperature, as the environmental humidity increases, the moisture absorption of the drug also increases. The co-processed excipients, with high proportions of MCC and L-HPC, exhibit strong hygroscopicity, making them prone to moisture absorption and affecting their stability. The moisture absorption equilibrium curve for the co-processed excipients under different humidity conditions was plotted using Origin 9.1 (Figure 3b), revealing that the critical relative humidity (CRH) of the co-processed excipient is 84.67%. This indicates that when the RH of the environment surpasses this threshold, the co-processed excipient absorbs a significant amount of water from the surroundings, altering its physicochemical properties and thereby impacting its stability. Therefore, to maintain stable physicochemical properties, the humidity of the packaging and storage environment for the co-processed excipients should be kept below 84.6%.

### 3.4. Surface Morphology, Physical States, and Particle Size

**Figure 4:**
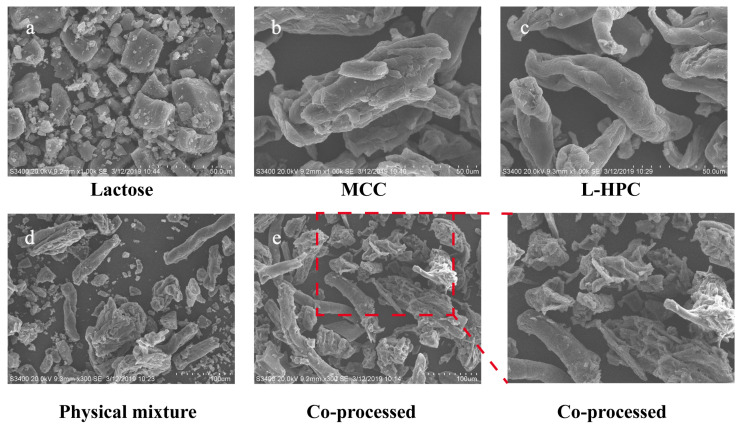
SEM images of different excipients. ((**a**): Lactose, bar = 50 μm; (**b**): MCC, bar = 50 μm; (**c**): L-HPC, bar = 50 μm; (**d**): physical mixture, bar = 100 μm; (**e**): co-processed excipient, bar = 100 μm).

The surface morphology of the material was analyzed using SEM, with the findings presented in Figure 4. In comparison to the individual excipients and their physical mixture, the particle shape and surface morphology of the co-processed excipients exhibited notable differences. The co-processed particles formed a dense and uniform combination of MCC, L-HPC, and lactose, whereas the materials in the physical mixture were relatively dispersed with poor mixing uniformity. Additionally, the surface of the co-processed excipient particles was rough, featuring adhered recrystallized lactose particles, which enhances the fluidity of the co-processed excipients. These structural characteristics resulted in high surface porosity and increased surface area, which significantly improved compressibility and further accelerated swelling and disintegration processes.

**Figure 5:**
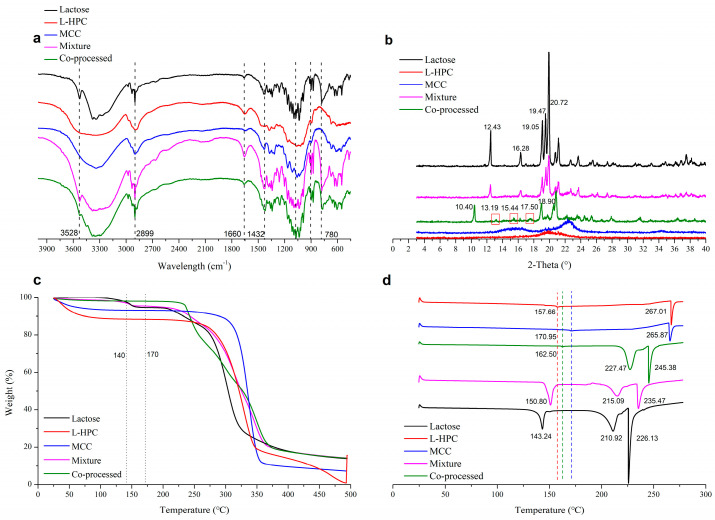
Physical characterization of various excipients in IR spectrum (**a**), XRD spectrum (**b**), TGA (**c**), and DSC thermogram (**d**).

In the infrared spectrum of the co-processed excipients (Figure 5a), no new characteristic peaks appeared compared to the physical mixture excipients. This indicated that the co-processed excipients did not form new chemical bonds, meaning no new chemical substances were introduced or produced during the co-processing. The XRD spectrum (Figure 5b) showed that L-HPC and MCC exhibited no characteristic peaks, while lactose displayed peaks (2θ) at 12.43° and 16.28°, which were distinct for α-lactose [36]. The physical mixture materials showed the same α-lactose signals. However, the co-processed excipients exhibited characteristic peaks at 10.40°, 13.19°, and 17.50°, with changes in the characteristic peaks of α-lactose. Among these peaks, the peak at 10.40° is characteristic of β-lactose [37]. This indicated that during co-processing, α-lactose monohydrate underwent a change in optical rotation, converting to β-lactose. Compared to the physical mixture, aside from the crystallization peaks resulting from lactose rotation, no other crystallization peaks of the co-processed excipients appeared or disappeared. Therefore, no chemical changes occurred during co-processing, and no new chemical substances were produced, meeting the requirements of the co-processed excipients.

In the TGA thermogram (Figure 5c), the co-processed excipients composed of lactose, MCC, and L-HPC exhibited a different thermal decomposition behavior compared to the physical mixture excipients. The co-processed excipients were more stable than the physical mixture in the range of 120–200 °C, with the thermal decomposition temperature of the co-processed excipients occurring at 281.27 °C (Figure S3E). In contrast, the physical mixture (Figure S3D) showed a similar thermal decomposition profile to α-lactose (Figure S3B). The physical mixture underwent three decomposition stages: loss of crystal water from α-lactose monohydrate at 141.08 °C, decomposition of lactose at 226.04 °C, and decomposition of both lactose and cellulose at 301.86 °C. The co-processed excipients did not show the characteristic peak around 141 °C, due to the change in the crystal form of lactose during processing, but displayed a distinct decomposition peak with an onset at 281.27 °C.

In the DSC thermogram (Figure 5d), characteristic cellulose melting peaks for L-HPC and MCC were observed at 267.01 °C and 265.87 °C, respectively, with binding water loss at 157.66 °C and 170.95 °C, respectively. Lactose showed a water loss peak at 143.24 °C and a melting peak at 210.92 °C. The physical mixture displayed the dehydration peak of lactose at 150.80 °C and melting peaks for lactose and cellulose at 215.09 °C and 234.47 °C, respectively. The dehydration peak of the co-processed excipients, originating from cellulose, occurs at 162.50 °C due to the conversion of α-lactose monohydrate to β-anhydrous lactose during co-processing. Melting peaks of β-anhydrous lactose (227.47 °C) and cellulose (245.38 °C) observed in the co-processed excipients shifted to higher temperatures compared with physical mixture (215.09 °C and 235.47 °C, respectively). This may be due to the increased stability of the excipient molecules formed during co-processing, enhancing the thermal stability of the co-processed excipients. Compared with the physical mixture, no new crystallization or melting peaks appeared in the co-processed excipients which proved that no new chemical was produced during the co-processing.

### 3.5. In Vitro Dissolution

**Figure 6:**
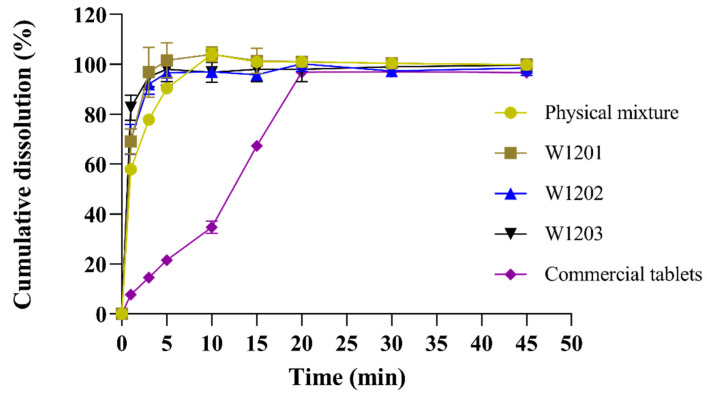
Release curves of MCP ODTs and metoclopramide tablets (*n* = 6).

The MCP ODTs were prepared using the optimal co-processed excipients formulation, and their dissolution rate was compared with ODTs prepared from a common physical mixture and commercially available metoclopramide tablets (Reglan^®^). The experimental results showed that that the dissolution rate was highest for the optimized co-processed excipients preparation, followed by the general physical mixture preparation, and lowest for the commercial tablet (Figure 6). The three batches of MCP ODTs prepared with the co-processed excipients exhibited a stable dissolution rate with no significant differences between them.

For MCP ODTs, L-HPC has strong hygroscopic properties, allowing it to swell with water. When combined with MCC, the porous structure of MCC is linearly arranged through disorder and plastic deformation. This structure allows water molecules to enter the tablet, breaking the hydrogen bonds between crystallites, leading to rapid disintegration. Additionally, the co-processing process resulted in a tight combination of MCC, L-HPC, and lactose, creating a large surface porosity and enhancing water swelling properties. This promoted disintegration and facilitated drug dissolution, making the co-processing method suitable for the formulation of orally dispersible and fast immediate-release tablets.

### 3.6. In Vivo Pharmacokinetics Study

**Figure 7:**
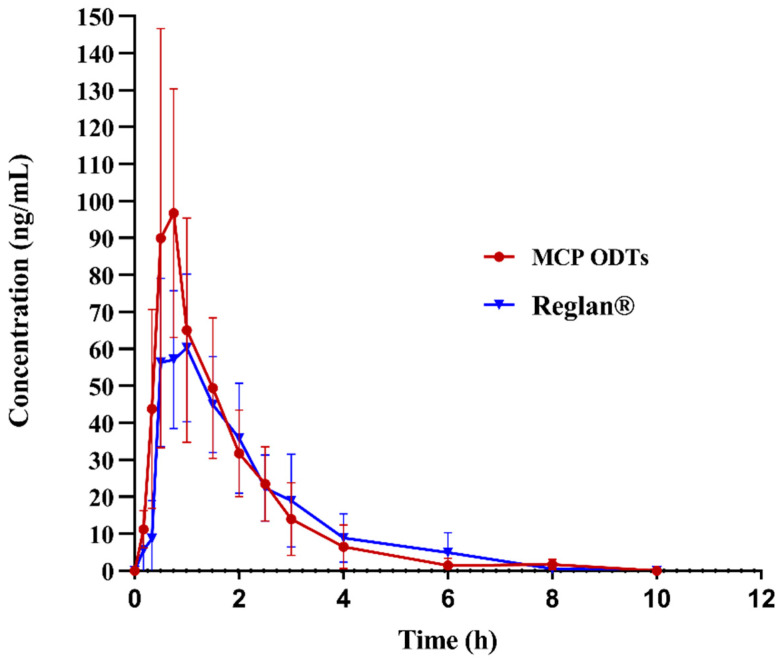
Mean plasma concentration–time profile of metoclopramide after administration of 10 mg control tablets and MCP ODTs in 6 beagle dogs.

In this study, non-compartmental model fitting was conducted using Winnolin7.0 software to analyze and calculate the main pharmacokinetic parameters of metoclopramide following a single 10 mg oral dose under fasting conditions. The average drug plasma concentration–time curves were illustrated in Figure 7, and the corresponding pharmacokinetic parameters were presented in Table 9, Table 10 and Table 11. The maximum plasma concentration (C_max_) differed significantly between MCP ODTs (121.99 ± 53.82 ng/mL) and Reglan^®^ (95.94 ± 51.08 ng/mL), with respective times to reach maximum concentration (T_max_) of 0.92 ± 0.36 h and 1.17 ± 0.43 h. Specifically, C_max_ was notably higher for MCP ODTs compared to conventional tablets, while T_max_ was shorter.

AUC_0-t_ and AUC_0-∞_ were logarithmically transformed and calculated using Winnolin7.0 software. The 90% confidence interval (CI) acceptance criteria for the test/reference ratio of AUC_0-t_, AUC_INF_obs_, and C_max_ are 80.00–125.00% [38]. According to Table 10, the 90% CIs for AUC_0-t_ and AUC_INF_obs_ were 93.58–124.51% and 94.56–126.38%, respectively. AUC_0-t_ fell within the confidence interval of 80–125%, while AUC_INF_ was almost within 80–125%. These results indicated that the bioequivalence evaluation using AUC_0-t_ as the criterion suggests equivalence between the MCP ODTs and the control preparation. However, the 90% CI for C_max_ after logarithmic transformation was 97.86–180.90%, which did not fall within the 80–125% confidence interval. Therefore, the bioequivalence evaluation using C_max_ as the criterion suggests non-equivalence between MCP ODTs and Reglan^®^. From Table 11, the probability (*p* = 0.1967) indicated no significant difference in T_max_ among different administration preparations, suggesting equivalence in T_max_. This means that MCP ODTs exhibited a similar AUC and T_max_ compared to Reglan^®^, but the C_max_ of MCP ODTs was higher than that of Reglan^®^ tablets. This suggests that the ODTs dissolve and absorb quickly, reaching C_max_ rapidly to achieve therapeutic effects. Future studies regarding validation of MCP ODTs efficiency should be performed, as the relative error due to limited sample size may affect data accuracy in the current study.

## 4. Conclusions

In this study, the lactose/L-HPC/MCC triple-based co-processed excipients were developed using a straightforward solvent evaporation method. The optimized formulation of MCP ODTs was prepared using co-processed excipients designed through single-factor and Box–Behnken experiments. The optimal ratios were determined as follows: water: L-HPC (*w*/*w*) = 17.26: 1, MCC: L-HPC (*w*/*w*) = 2.79: 1, and lactose: L-HPC (*w*/*w*) = 4.54: 1. The results demonstrated that particles formed from solvent evaporation using co-processed excipients exhibited superior flowability and compatibility compared to physical mixtures. Tablets compressed with these particles showed significantly reduced disintegration times to less than 25 s and achieved complete dissolution within 5 min. Pharmacokinetic studies indicated that MCP ODTs had a higher and faster drug absorption and were bioequivalent to the marketed products regarding AUC and T_max_. In conclusion, the MCP ODTs made from lactose/L-HPC/MCC triple-based co-processed excipients demonstrated significant potential in enhancing tablet performance in terms of disintegration, drug release, bioequivalence, and absorption rate, making them a promising dosage form for the pharmaceutical industry.

## Figures and Tables

**Table 1:** Powder fluidity of co-processed excipients prepared with different filler agents.

Filler Agents	Angle of Repose (°)	Bulk Density (g/mL)	Tap Density (g/mL)	Hausner Ratio	Carr’s Index (%)
Lactose	37.09 ± 2.96	0.3653 ± 0.008	0.4823 ± 0.013	1.32 ± 0.075	24.26 ± 0.54
Mannitol	39.11 ± 0.83	0.3236 ± 0.013	0.5153 ± 0.021	1.59 ± 0.061	37.20 ± 1.42
Maltitol	46.68 ± 2.75	0.3200 ± 0.018	0.5247 ± 0.017	1.64 ± 0.068	39.01 ± 0.87
Sorbitol	44.42 ± 1.97	0.2566 ± 0.011	0.3708 ± 0.015	1.45 ± 0.081	30.80 ± 0.83

Data presented are as the mean ± SD of three independent experiments.

**Table 2:** Powder fluidity of co-processed excipients prepared with different disintegrating agents.

Disintegrating Agents	Angle of Repose (°)	Bulk Density (g/mL)	Tap Density (g/mL)	Hausner Ratio	Carr’s Index (%)
L-HPC (LH22)	35.67 ± 2.21	0.4348 ± 0.014	0.5685 ± 0.016	1.31 ± 0.061	23.50 ± 0.75
L-HPC (LH21)	34.57 ± 1.91	0.3751 ± 0.023	0.4716 ± 0.017	1.26 ± 0.058	20.46 ± 1.00
L-HPC (LB)	42.67 ± 0.50	0.4171 ± 0.018	0.6107 ± 0.011	1.46 ± 0.047	31.70 ± 0.91
CMS-Na (1)	37.06 ± 3.33	0.5195 ± 0.021	0.7050 ± 0.013	1.36 ± 0.089	26.31 ± 1.48
CMS-Na (2)	43.51 ± 1.48	0.5538 ± 0.011	0.7384 ± 0.022	1.33 ± 0.062	25.00 ± 0.81

Notes: CMS-Na (1): rapid collapse; CMS-Na (2): normal type. Data presented are as the mean ± SD of three independent experiments.

**Table 3:** Powder fluidity of co-processed excipients prepared with different heat treatment times at 80 °C.

Added Materials	Added Time (h)	Angle of Repose (°)	Bulk Density (g/mL)	Tap Density (g/mL)	Hausner Ratio	Carr’s Index (%)
L-HPC	0.25	34.26 ± 0.43	0.4562 ± 0.016	0.5977 ± 0.018	1.31 ± 0.066	23.68 ± 0.68
	1	37.79 ± 0.82	0.4803 ± 0.024	0.6403 ± 0.011	1.33 ± 0.041	24.98 ± 0.81
	2	36.40 ± 1.43	0.5040 ± 0.018	0.6719 ± 0.021	1.33 ± 0.058	24.98 ± 0.97
	3	37.62 ± 1.11	0.4640 ± 0.027	0.6496 ± 0.027	1.40 ± 0.049	28.57 ± 0.89
	4	41.67 ± 1.55	0.4935 ± 0.031	0.6807 ± 0.029	1.38 ± 0.083	27.50 ± 1.30
	6	42.81 ± 1.71	0.4060 ± 0.025	0.6091 ± 0.032	1.50 ± 0.053	33.34 ± 1.52
Lactose	0.5	39.76 ± 1.52	0.4775 ± 0.019	0.6686 ± 0.022	1.40 ± 0.062	28.57 ± 0.71
	1	35.77 ± 0.96	0.4834 ± 0.007	0.6387 ± 0.011	1.32 ± 0.087	24.32 ± 0.98
	1.5	33.84 ± 0.56	0.5148 ± 0.015	0.6642 ± 0.018	1.29 ± 0.065	22.50 ± 0.83
	2	36.44 ± 1.06	0.4948 ± 0.026	0.6385 ± 0.022	1.29 ± 0.094	22.50 ± 0.79
	2.5	39.94 ± 1.63	0.5087 ± 0.023	0.6783 ± 0.021	1.33 ± 0.053	25.00 ± 0.97
	3	32.85 ± 2.61	0.5339 ± 0.033	0.6811 ± 0.028	1.28 ± 0.079	21.61 ± 1.39
	4	28.44 ± 0.32	0.4748 ± 0.015	0.6280 ± 0.017	1.32 ± 0.058	24.39 ± 0.96
MCC	3	34.42 ± 0.37	0.4631 ± 0.018	0.5975 ± 0.015	1.29 ± 0.075	22.50 ± 0.74
	6	37.36 ± 2.00	0.4965 ± 0.028	0.6262 ± 0.029	1.26 ± 0.066	20.71 ± 0.99
	9	45.28 ± 0.23	0.4846 ± 0.015	0.6576 ± 0.016	1.36 ± 0.058	26.32 ± 0.87
	18	40.51 ± 0.35	0.4709 ± 0.021	0.6076 ± 0.024	1.29 ± 0.083	22.50 ± 1.55

Data presented are as the mean ± SD of three independent experiments.

**Table 4:** Powder fluidity of co-processed excipients prepared with different heat treatment temperatures for 3 h.

Added Materials	Added Temperature (°C)	Angle of Repose (°)	Bulk Density (g/mL)	Tap Density (g/mL)	Hausner Ratio	Carr’s Index (%)
L-HPC	RT	43.47 ± 2.53	0.5653 ± 0.035	0.7828 ± 0.017	1.38 ± 0.083	27.78 ± 0.85
	40	43.20 ± 0.17	0.4291 ± 0.011	0.6007 ± 0.013	1.40 ± 0.069	28.57 ± 0.84
	50	41.63 ± 1.48	0.4405 ± 0.028	0.6075 ± 0.022	1.38 ± 0.047	27.49 ± 0.93
	60	34.11 ± 3.05	0.4863 ± 0.043	0.6383 ± 0.035	1.31 ± 0.055	23.81 ± 0.77
	70	40.65 ± 2.50	0.4682 ± 0.037	0.6399 ± 0.036	1.37 ± 0.072	26.83 ± 1.69
	80	36.74 ± 0.63	0.5196 ± 0.016	0.6726 ± 0.018	1.29 ± 0.093	22.75 ± 0.90
	90	36.98 ± 0.02	0.4795 ± 0.009	0.6293 ± 0.012	1.31 ± 0.047	23.80 ± 1.43
Lactose	RT	41.16 ± 2.07	0.5255 ± 0.026	0.7591 ± 0.025	1.44 ± 0.052	30.77 ± 0.88
	40	43.31 ± 0.46	0.4952 ± 0.022	0.6933 ± 0.021	1.40 ± 0.061	28.57 ± 0.96
	50	41.56 ± 0.64	0.5019 ± 0.016	0.6849 ± 0.013	1.36 ± 0.077	26.72 ± 0.72
	60	37.66 ± 0.99	0.5211 ± 0.027	0.7071 ± 0.021	1.36 ± 0.059	26.30 ± 1.28
	70	40.31 ± 1.05	0.5239 ± 0.022	0.6811 ± 0.028	1.30 ± 0.060	23.08 ± 1.13
	80	32.85 ± 3.62	0.5479 ± 0.029	0.6849 ± 0.025	1.25 ± 0.064	20.00 ± 0.99
	90	42.24 ± 2.06	0.5071 ± 0.031	0.7136 ± 0.036	1.41 ± 0.073	28.94 ± 0.83
MCC	RT	36.09 ± 1.12	0.4884 ± 0.018	0.6411 ± 0.019	1.31 ± 0.059	23.81 ± 1.21
	40	37.50 ± 0.71	0.485 ± 0.023	0.6466 ± 0.026	1.33 ± 0.046	25.00 ± 0.55
	50	40.67 ± 1.00	0.4786 ± 0.017	0.6475 ± 0.016	1.35 ± 0.083	26.09 ± 0.89
	60	44.40 ± 0.75	0.453 ± 0.011	0.6098 ± 0.014	1.35 ± 0.070	25.71 ± 0.86

RT means room temperature, data presented are as the mean ± SD of three independent experiments.

**Table 5:** Process variables and levels in Box–Behnken design and experimental results.

Formulation Number	Water: L-HPC (g/g)	MCC: L-HPC (g/g)	Lactose: L-HPC (g/g)	Disintegrating Time (s)	Angle of Repose (°)	Carr’s Index (%)
1	20	3	4	19.54	36.92	20.83
2	30	3	2	40.66	39.95	28.57
3	20	3	4	22.28	38.61	23.53
4	10	3	6	25.95	38.46	18.75
5	20	2	6	26.64	41.30	20.83
6	10	2	4	20.85	41.41	25.00
7	20	3	4	21.59	37.55	21.05
8	10	4	4	31.32	42.27	30.77
9	20	4	6	35.49	43.18	25.00
10	30	3	6	44.12	40.44	35.71
11	20	3	4	20.64	37.83	23.53
12	30	4	4	41.41	41.69	29.41
13	20	2	2	33.31	40.37	26.67
14	20	3	4	22.93	37.53	18.75
15	10	3	2	25.04	40.03	28.57
16	30	2	4	38.14	38.66	30.77
17	20	4	2	38.94	40.93	33.33

**Table 6:** Model summary statistics.

Response	Source	Std. Dev.	R-Squared	
Disintegrating time (s)	Quadratic	2.35	0.9663	Suggested
Angle of repose (°)	Quadratic	1.03	0.8642	Suggested
Carr’s index (%)	Quadratic	0.029	0.8611	Suggested

**Table 7:** The powder fluidity and formulation disintegration time of optimized formulation.

Sample	Mean Angle of Repose (°)	Hausner Ratio	Carr’s Index (%)	Mean Disintegrating Time (s)
181201	34.98	1.23	18.75	23.06
181202	37.31	1.25	20.59	23.69
181203	37.21	1.23	21.94	21.53

Data presented are as the mean of three independent experiments.

**Table 8:** Powder fluidity of co-processed excipients.

Parameter	Value
Bulk density (g/mL)	0.60–0.63
Tab density (g/mL)	0.77–0.80
Hausner ratio	1.23
Carr’s index (%)	18.75
Angle of repose (°)	36.52

Data presented are as the mean of three independent experiments.

**Table 9:** Pharmacokinetic parameters of drug in beagle dogs after oral administration of MCP ODTs and Reglan^®^.

Parameters	Reglan^®^	MCP ODTs
T_1/2_ (h)	1.17 ± 0.43	0.92 ± 0.36
T_max_ (h)	0.75	1
*C*_max_ (ng/mL)	60.30 ± 51.08 *	96.74 ± 53.82 *
T_last_ (h)	7.33 ± 1.03	5.67 ± 1.97
AUC_0-t_ (h × ng/mL)	142.56 ± 54.72	152.39 ± 56.41
AUC_0-∞_ (h × ng/mL)	145.83 ± 57.29	157.85 ± 59.64
Vd_0-∞_ (L)	124.32 ± 37.89	92.49 ± 43.13
CL_0-∞_ (L/h)	82.61 ± 45.55	74.10 ± 34.79
MRT_0-t_	1.99 ± 0.58	1.50 ± 0.36
MRT_0-∞_	2.13 ± 0.70	1.65 ± 0.40

*n* = 6, ±standard deviation. * means significant difference and *p* < 0.05.

**Table 10:** Relative bioavailability.

Dependent	Units	Ratio_% Ref_	CI_90_Lower	CI_90_Upper
Ln (*C*_max_)	ng/mL	133.05	97.86	180.90
Ln (AUC_last_)	h × ng/mL	107.94	93.58	124.51
Ln (AUC_INF_obs_)	h × ng/mL	109.32	94.56	126.38

**Table 11:** Non-parametric test results of T_max_.

Test	*p* Value
Sequence	0.3758
Period	1
Form	0.1967

## Data Availability

Data are contained within the article and Supplementary Materials.

## Supplementary Materials

The following supporting information can be downloaded at https://www.mdpi.com/article/10.3390/pharmaceutics16070959/s1, Figure S1: Specificity spectrums of HPLC chromatogram analysis methodology; Figure S2: Calibration curve of metoclopramide in Beagle plasma; Figure S3: TGA thermogram of L-HPC, lactose, CMC, physical mixture and co-processed; Figure S4: DSC thermogram with enthalpy values of L-HPC, lactose, CMC, physical mixture and co-processed; Figure S5: Particle size and size distribution of co-processed excipients; Table S1: Varibles in Box -Behnken design for co-processed excipients; Table S2: Relative humidity (RH) of different saturated saline solution (at 25 °C); Table S3: Analysis of variance for regression model (Response: disintegrating time); Table S4: Analysis of variance for regression model (Response angle of repose); Table S5: Analysis of variance for regression model (Response Carr’s Index); Table S6: Results of method resolution of determination method of disintegration time; Table S7: Results of method correlation of determination method of disintegration time; Table S8: Result of method reproducibility of determination method of disintegration time.
