# Supplementary material for: A Novel Lactose/MCC/L-HPC Triple-Based Co-Processed Excipients with Improved Tableting Performance Designed for Metoclopramide Orally Disintegrating Tablets
Source: Pharmaceutics. 2024 Jul 19;16(7):959. doi: 10.3390/pharmaceutics16070959 (PMC11279886; doi:10.3390/pharmaceutics16070959)
Supplement: Supplementary file 1 [file pharmaceutics-16-00959-s001.zip › pharmaceutics-3086070-supplementary.pdf]

A Novel Lactose/MCC/L-HPC Triple-Based Co-Processed Excipients with Improved Tableting Performance Designed for Metoclopramide Orally Disintegrating Tablets

Xiaorong Dai[1], Jiamin Wang[2], Bo Yan[3], Qian Wang[2], Yan Shen[2], Yongkang Chen[1,*], Yu Tian[4, 5*]

[1] Department of Gastroenterology, Taixing People's Hospital, No.1 Changzheng Road, Taixing, Jiangsu, China

[2] Center for Research Development and Evaluation of Pharmaceutical Excipients and Generic Drugs, School of Pharmacy, China Pharmaceutical University, 24 Tong Jia Xiang, Nanjing 210009, China

[3] Department of Pharmacy, Taixing People's Hospital, No.1 Changzheng Road, Taixing, Jiangsu, China

[4] School of Medicine, Shanghai University, Shanghai, 200444, China

[5] Institute of Geriatrics (Shanghai University), Affiliated Nantong Hospital of Shanghai University (The Sixth People's Hospital of Nantong), School of Medicine, Shanghai University, Nantong, 201613, China

*Corresponding authors: E-mail: erdong321025@163.com (Y.C.), Shu_yutian@shu.edu.cn (Y.T.).

Table S1. Varibles in Box -Behnken design for co-processed excipients

Factors	levels and coded		
	-1	0	1
**Independent variables**			
X1:water:L -HPC(g/g)	10	20	30
X2:MCC:L -HPC(g/g)	2	3	4
X3: Lactose:L -HPC(g/g)	2	4	6
**Dependent variables**			
Y1:Disintegrating time(s)			Minimize
Y2:Angle of repose(°)			Minimize
Y3：Carr Index(%)			Minimize

Table S2. Relative humidity (RH) of different saturated saline solution (at 25 °C)

Saturated solution	CH_3COOK	$CaCl_2$	$NaBr_2$	NaCl	$(NH_4)_2SO_4$	$KHSO_4$	$ZnSO_4·7H_2O$	$Na_2HPO_3·12H_2O$
RH (%)	20	37	58	75	82	88	92	96

Table S3. Analysis of variance for regression model (Response: disintegrating time)

Sum of Square	Df	Mean Square	F value	P-value Prob>F	Significant
1107.0094	9	123.0010	22.2921	0.0002	**
467.6771	1	467.6771	84.7593	< 0.0001	**
99.5696	1	99.5696	18.0455	0.0038	**
4.0976	1	4.0976	0.7426	0.4174	
13.0171	1	13.0171	2.3591	0.1684	
1.6197	1	1.6197	0.2936	0.6048	
2.5961	1	2.5961	0.4705	0.5148	
148.6498	1	148.6498	26.9405	0.0013	**
131.8047	1	131.8047	23.8876	0.0018	**
183.7392	1	183.7392	33.2999	0.0007	**
38.6240	7	5.5177			
31.4288	3	10.4763	5.8241	0.0609	
7.1952	4	1.7988			
1145.6333	16				
0.9663					

Levels of significance are denoted as * $p < 0.05$, ** $p < 0.01$.

Table S4. Analysis of variance for regression model (Response angle of repose)

Source	Sum of Square	Df	Mean Square	F value	P-value Prob>F	Significant
Model（2）	47.4958	9	5.2773	4.9510	0.0233	*
X_1	0.2592	1	0.2592	0.2432	0.6370	
X_2	5.0192	1	5.0192	4.7089	0.0666	
X_3	0.5495	1	0.5495	0.5155	0.4960	
X_1X_2	1.1736	1	1.1736	1.1011	0.3289	
X_1X_3	1.0540	1	1.0540	0.9889	0.3531	
X_2X_3	0.4378	1	0.4378	0.4107	0.5420	
X_1^2	2.6729	1	2.6729	2.5076	0.1573	
X_2^2	26.8288	1	26.8288	25.1701	0.0015	**
X_3^2	6.4142	1	6.4142	6.0176	0.0439	*
Residual	7.4613	7	1.0659			
Lack of Fit	5.9551	3	1.9850	5.2717	0.0711	
Pure Error	1.5062	4	0.3765			
Cor Total	54.9571	16				
R^2	0.8642					

Levels of significance are denoted as * $p < 0.05$, ** $p < 0.01$.

Table S5. Analysis of variance for regression model (Response Carr's Index)

Source	Sum of Square	Df	Mean Square	F value	P-value Prob>F	Significant
Model（3）	0.0358	9	0.0040	4.8229	0.0250	*
X_1	0.0057	1	0.0057	6.9192	0.0339	*
X_2	0.0029	1	0.0029	3.5193	0.1028	
X_3	0.0035	1	0.0035	4.2969	0.0769	
X_1X_2	0.0013	1	0.0013	1.5382	0.2548	
X_1X_3	0.0072	1	0.0072	8.7157	0.0213	*
X_2X_3	0.0002	1	0.0002	0.1893	0.6766	
X_1^2	0.0083	1	0.0083	10.0826	0.0156	*
X_2^2	0.0038	1	0.0038	4.5985	0.0692	
X_3^2	0.0015	1	0.0015	1.8741	0.2133	
Residual	0.0058	7	0.0008			
Lack of Fit	0.0041	3	0.0014	3.3541	0.1365	
Pure Error	0.0016	4	0.0004			
Cor Total	0.0416	16				
R^2	0.8611					

Levels of significance are denoted as * $p < 0.05$.

Table S6. Results of method resolution of determination method of disintegration time

Prescription	Disintegration time (s, n=6)							
number	1	2	3	4	5	6	Mean	RSD/%
1	29	27	26	22	26	27	26	8.6
2	21	17	21	19	19	21	20	8.3
3	22	23	25	24	25	25	24	5.2
4	19	21	17	17	20	18	19	8.0
5	30	34	32	33	28	30	31	7.2
6	55	57	50	58	60	60	57	6.6

Table S7. Results of method correlation of determination method of disintegration time

Method	Prescription	Disintegration time (s, n=6)							
	number	1	2	3	4	5	6	Mean	RSD/%
Homemade device	1	25	27	26	22	26	27	26	3.0
	3	22	23	25	24	25	25	24	5.2
	5	30	34	32	33	28	30	31	7.2
Volunteer oral test	1	26	27	25	23	26	25	25	5.5
	3	21	22	26	25	24	25	24	8.1
	5	29	33	32	34	30	29	31	6.9

Table S8. Result of method reproducibility of determination method of disintegration time

Number	Disintegration time (s, n=6)							
	1	2	3	4	5	6	Mean	RSD/%
1	17	18	20	21	20	21	19	8.5
2	20	19	18	20	20	20	20	4.1
3	18	18	19	20	20	18	19	4.6

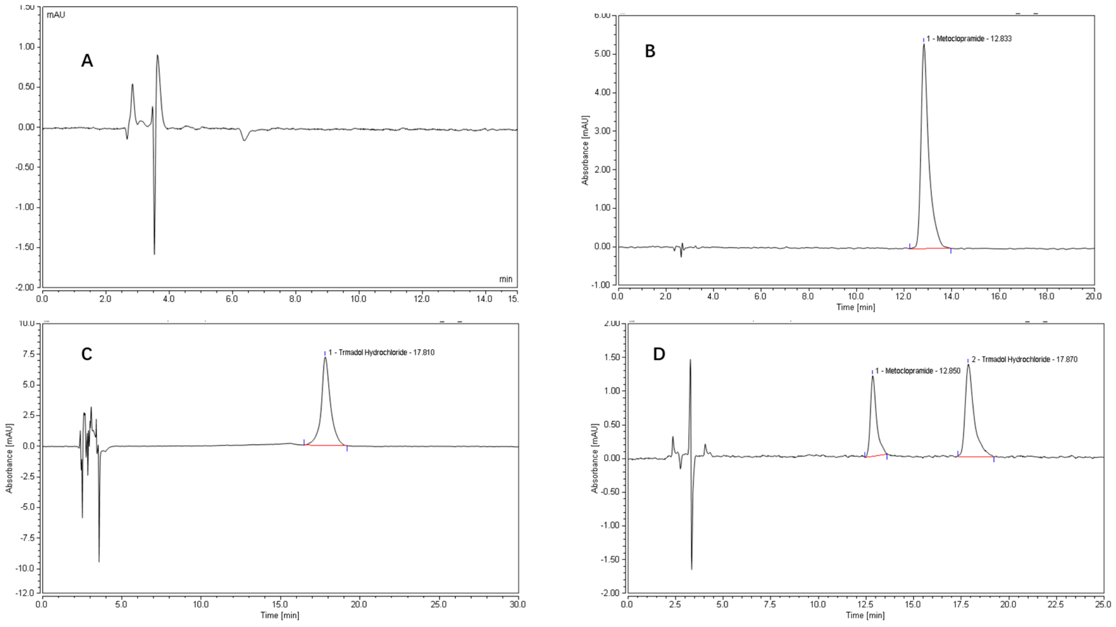

Figure S1. Specificity spectrums of HPLC chromatogram analysis methodology. A : Black Plasma; B : Black Plasma + Metoclopramide; C : Black Plasma + Internal standard; D : Plasma sample + Metoclopramide +Internal standard.

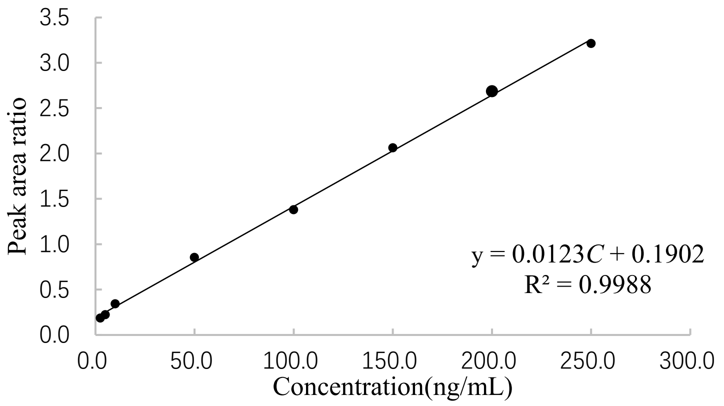

Figure S2 Calibration curve of metoclopramide in Beagle plasma

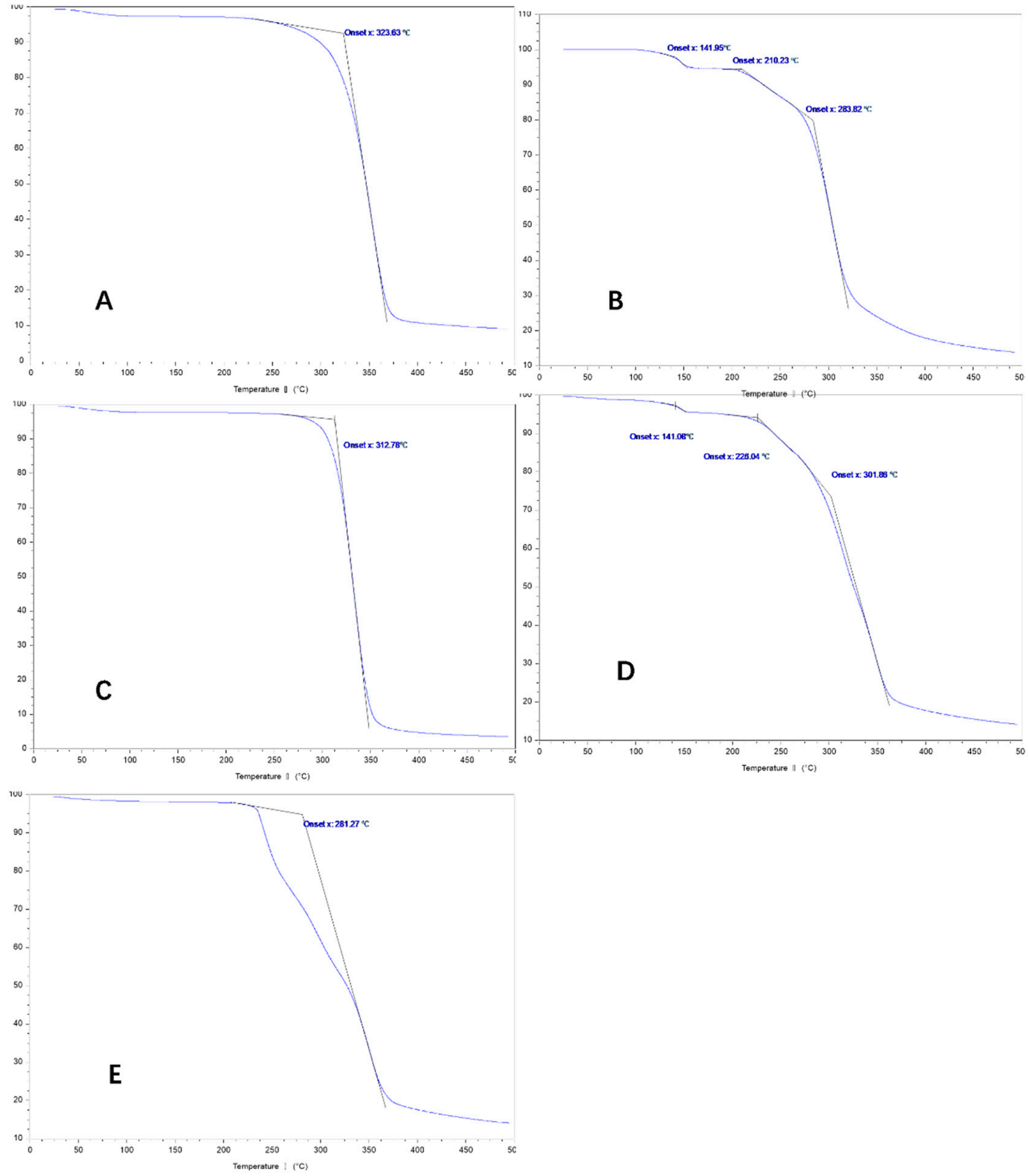

Figure S3. TGA thermogram of L-HPC (A), lactose (B), CMC (C), physical mixture (D) and co-processed (E).

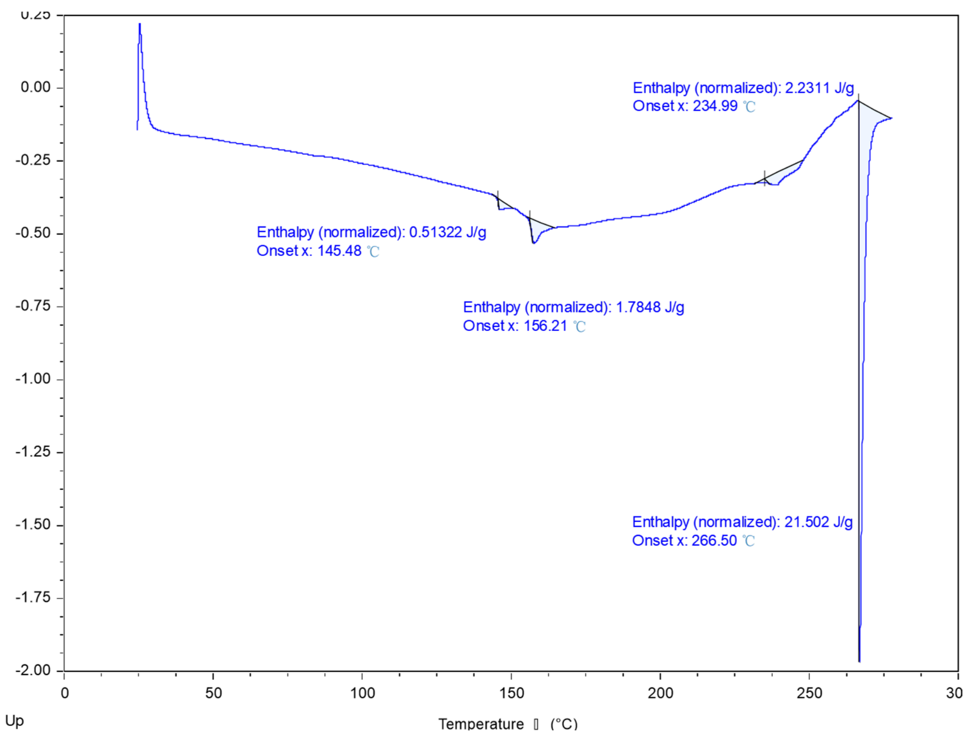

Figure S4-A

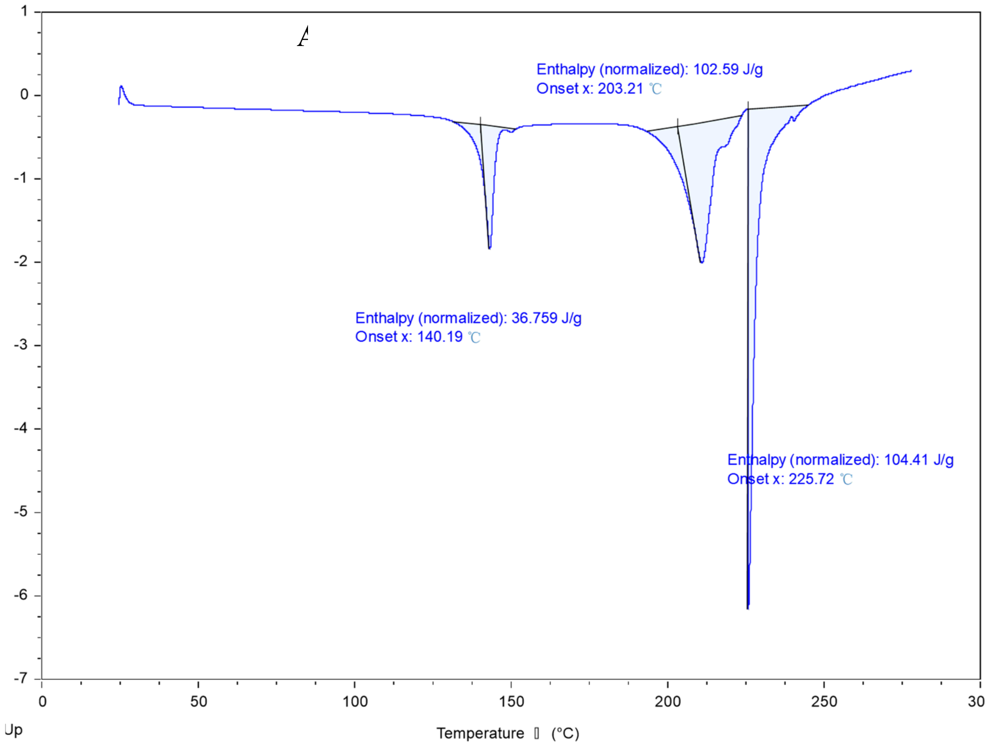

Figure S4-B

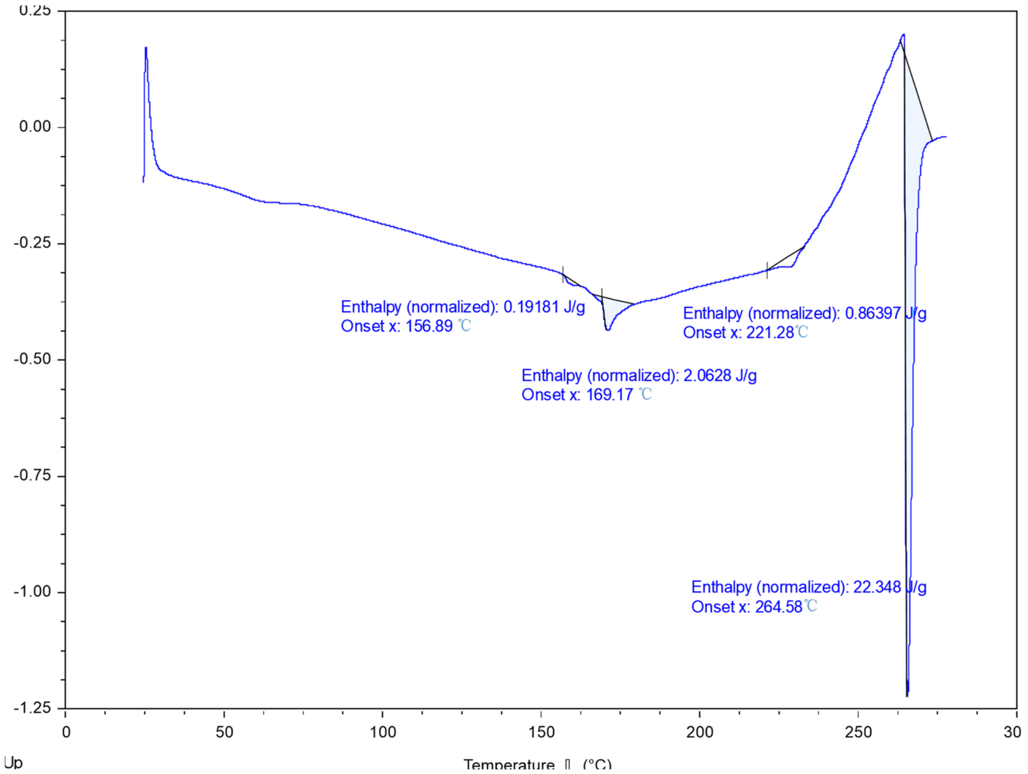

Figure S4-C

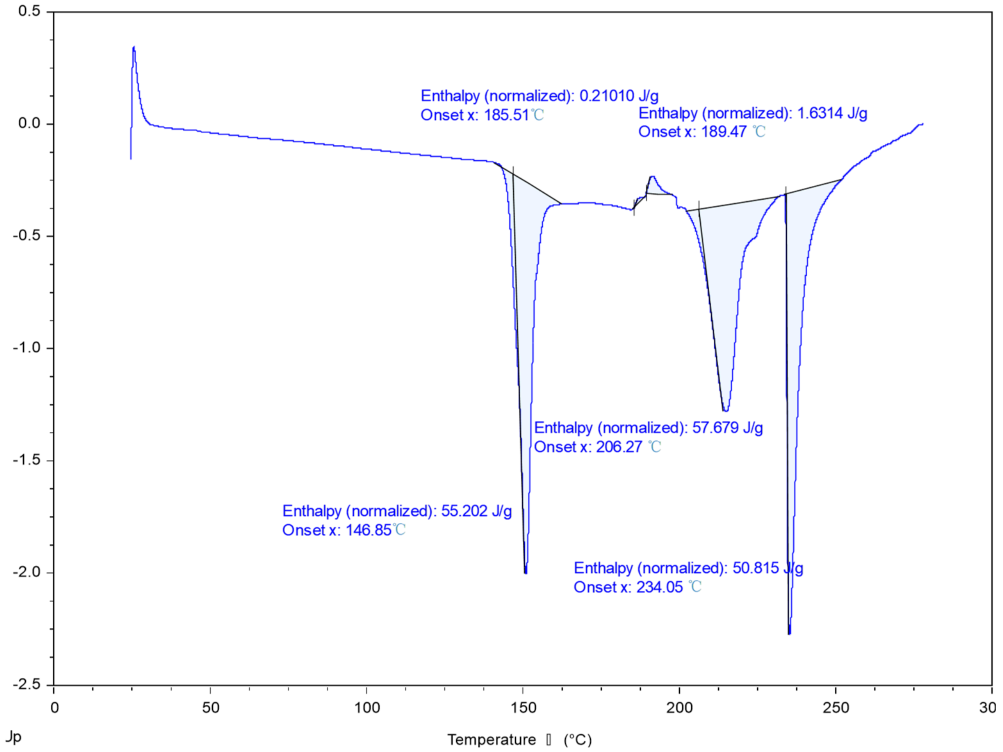

Figure S4-D

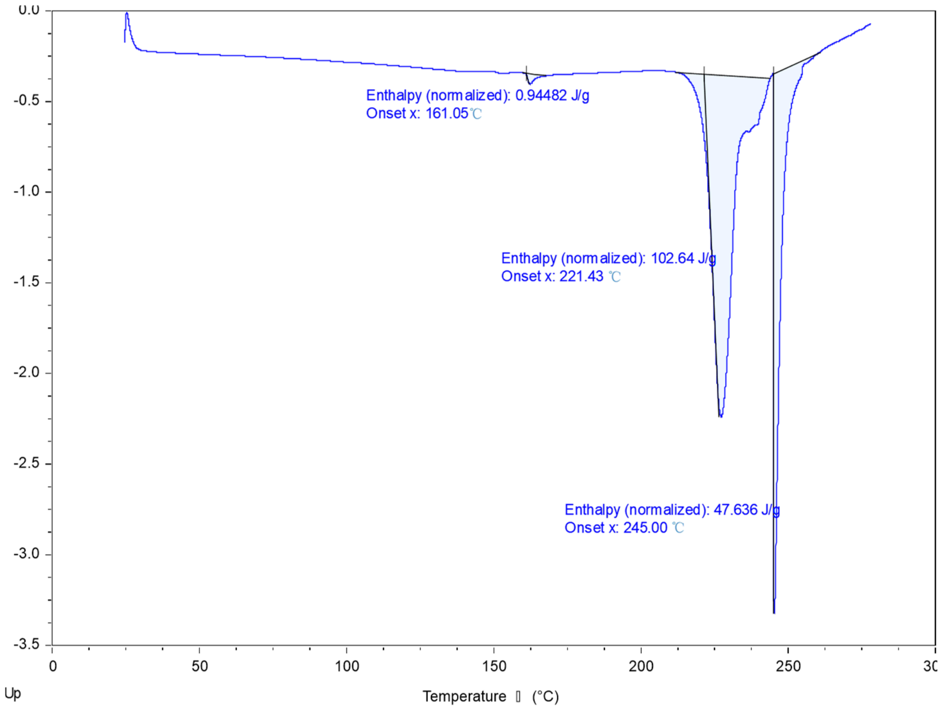

Figure S4-E

Figure S4. DSC thermogram with enthalpy values of L-HPC (A), lactose (B), CMC (C), physical mixture (D) and co-processed (E).

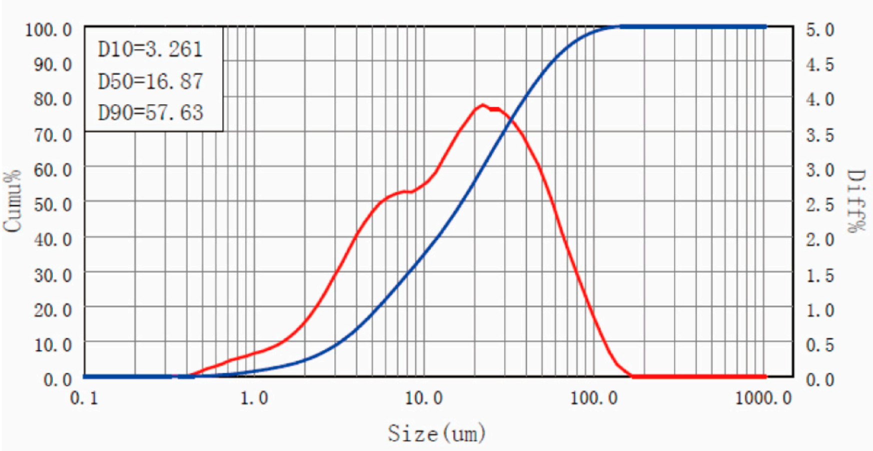

Particle size µm	Content %
0.500	0.09
1.000	1.46
2.000	4.68
5.000	18.03
10.00	34.77
20.00	55.83
45.00	83.55
75.00	95.07
100.0	98.48
200.0	100.00

Figure S5. Particle size and size distribution of co-processed excipients.
